# Track more, drop less: dual dimensions of usefulness and configurational pathways to sport wearable usage intention for digital public health

**DOI:** 10.3389/fpubh.2026.1869828

**Published:** 2026-07-15

**Authors:** Hyun Byun, Junwoo Lee, Dong-kyu Kim

**Affiliations:** 1 Sports Science Research Institute, Seoul National University of Science & Technology, Seoul, Republic of Korea; 2 Department of Golf Industry, Hoseo University, Chungcheongnam-do, Republic of Korea; 3 Department of Sport Industry Studies, Soonchunhyang University, Chungcheongnam-do, Republic of Korea

**Keywords:** exercise usefulness, fsQCA, functional usefulness, intention to use, perceived value, PLS-SEM, sport wearable devices

## Abstract

**Introduction:**

Physical inactivity is a major global public health challenge, and sport wearable devices have emerged as promising digital health tools. Despite high initial adoption rates, long-term adoption remains limited. This study examines whether distinguishing exercise usefulness (EU) from functional usefulness (FU) provides a more nuanced explanation of sport wearable usage intention.

**Methods:**

Extending the Technology Acceptance Model, perceived usefulness was reconceptualized as two distinct constructs, exercise usefulness and functional usefulness, alongside perceived value (PV) and intention to use (IU). Survey data from 322 Korean adults with prior sport wearable device experience were analyzed using partial least squares structural equation modeling (PLS-SEM) and fuzzy-set qualitative comparative analysis (fsQCA).

**Results:**

Both EU and FU were positively associated with PV and IU. FU demonstrated a stronger positive association with PV, whereas EU exerted a stronger direct association with IU. PV was positively associated with IU, and a value-mediated indirect effect was supported for FU but not for EU, indicating distinct pathways to adoption intention. The fsQCA identified five configurational pathways demonstrating equifinality and causal asymmetry, with functional usefulness emerging as the most consistently present condition.

**Discussion:**

Exercise usefulness and functional usefulness influence adoption intention through structurally distinct pathways, and high intention to use can emerge from multiple alternative configurations. These findings provide practical implications for the design and promotion of sport wearable technologies while highlighting important limitations regarding generalizability beyond Korean adults with prior sport wearable device experience.

## 1 Introduction

Physical inactivity remains a major global public health concern. The World Health Organization (WHO) estimates that approximately 27% of adults worldwide fail to meet recommended physical activity levels, contributing substantially to the burden of chronic diseases such as cardiovascular disease, type 2 diabetes, obesity, and depression (1). In South Korea, the Korea Disease Control and Prevention Agency (KDCA) reported that 58.1% of adults are physically inactive, a prevalence nearly twice the global average (2). In response to this challenge, sport wearable devices have gained increasing attention as promising digital health tools for promoting physical activity. Devices such as smartwatches, fitness bands, and sport trackers collect and analyze real-time physiological and behavioral data, including physical activity, heart rate, calorie expenditure, and sleep patterns. They also provide personalized feedback that may encourage behavior change and support healthier lifestyles (3). Unlike traditional interventions, which often require discrete episodes of engagement, wearables embed monitoring into everyday routines enabling ongoing monitoring and greater integration into everyday routines. As a result, they have considerable potential as scalable tools for population-level health promotion (4).

The growing relevance of wearable technologies is reflected in both market expansion and public health initiatives. IDC (5) reported that global wearable device shipments reached approximately 506 million units in 2023 and are expected to grow at a compound annual growth rate of 3.6% between 2024 and 2028. Wrist-worn devices account for more than half of total market revenue (6). At the same time, governments are increasingly integrating wearable technologies with artificial intelligence (AI) and the Internet of Things (IoT) to support large-scale health monitoring systems, particularly for aging populations (7). Despite rapid market growth and widespread adoption, continued adoption of wearable devices remains a challenge. Previous studies indicate that approximately 10% of users discontinue use within 3 months, while nearly 30% lose interest within the first month (8). Importantly, device abandonment cannot be explained solely by technological limitations. Instead, discontinuation appears to result from multiple factors, including user perceptions, motivations, and experiences. From a public health perspective, this issue is particularly important because the effectiveness of wearable technologies depends not only on initial adoption but also on continued engagement. Without sustained intention to use, the potential health benefits of wearable devices may not be fully realized (3). Consequently, identifying the factors that promote continued intention to use has become an important research objective in digital public health research.

Previous research has revealed a notable inconsistency. Users of technologically advanced wearable devices do not necessarily exhibit higher levels of exercise adherence (9, 10). In contrast, individuals who perceive meaningful improvements in exercise performance often maintain device usage even when device functionality is relatively limited (11, 12). These findings suggest that usefulness in the context of sport wearable devices may consist of multiple dimensions rather than a single construct. Therefore, a more refined conceptualization of usefulness is needed to explain how different forms of utility contribute to intention to use within the context of sport wearable technologies. Most previous studies have relied on the Technology Acceptance Model (TAM) to explain wearable device adoption (13). Within TAM, perceived usefulness is considered a key determinant of technology acceptance and behavioral intention (14) and has consistently been identified as one of the strongest predictors of intention to use in information systems and digital health research (15). However, treating usefulness as a unidimensional construct may be insufficient in the context of sport wearable technologies. Unlike conventional information systems, wearable devices directly support users’ physical activity and exercise behaviors. On one hand, they provide exercise-related benefits by facilitating goal attainment, strengthening self-efficacy, and encouraging behavior change (16). On the other hand, they offer functional benefits through technical attributes such as measurement accuracy, interface usability, and data reliability (17).

Despite this distinction, previous TAM-based studies on sport wearable devices [e.g., (18, 19)] have generally treated usefulness as a single construct. As a result, the potentially different roles of exercise-related and functional benefits remain underexplored. In addition, most prior studies have employed symmetric analytical approaches, such as structural equation modeling (SEM), which focus on estimating linear net effects among variables (20). Although these approaches are valuable for theory testing, they are less effective in capturing configurational causality. In particular, they cannot adequately explain equifinality, where different combinations of conditions lead to the same outcome, or causal asymmetry, where the causes of an outcome differ from the causes of its absence (21, 22). For example, high intention to use may emerge when users perceive strong exercise benefits despite limited functional convenience, whereas in other cases favorable functional perceptions may compensate for weaker exercise-related benefits (23). Accordingly, this study integrates PLS-SEM and fsQCA to examine both the net effects and configurational pathways through which exercise usefulness and functional usefulness are associated with intention to use sport wearable devices (24).

### 1.1 Objectives and approach

To address these theoretical and methodological gaps, this study makes three contributions. First, it distinguishes between exercise usefulness and functional usefulness as conceptually distinct forms of usefulness in the context of sport wearable technologies. This distinction provides a more nuanced understanding of how different forms of usefulness are associated with users’ evaluations and intentions. Second, the study adopts a mixed-method approach that combines partial least squares structural equation modeling (PLS-SEM) and fuzzy-set qualitative comparative analysis (fsQCA). PLS-SEM is well suited for prediction-oriented research and theory development (25), whereas fsQCA is particularly useful for identifying multiple configurations associated with the same outcome (20, 26). Together, these methods provide complementary insights into both symmetric and asymmetric patterns associated with intention to use sport wearable devices. Third, the study examines the mediating role of perceived value in the relationships among exercise usefulness, functional usefulness, and intention to use. Perceived value refers to consumers’ overall assessment of a product or service based on a comparison between perceived benefits and costs (27, 28). Examining this mediating mechanism provides additional insight into how exercise usefulness and functional usefulness are associated with intention to use through value-based evaluations.

### 1.2 Research questions and scholarly contributions

Building on the preceding discussion, this study addresses the following research questions:

RQ1: Are exercise usefulness and functional usefulness associated with intention to use sport wearable devices, and do their associations differ in magnitude?

RQ2: Does perceived value mediate the relationships between exercise usefulness, functional usefulness, and intention to use?

RQ3: Which configurations of conditions are sufficient for high intention to use?

This study contributes to the literature in three ways. First, it extends the Technology Acceptance Model (TAM) by distinguishing between exercise usefulness and functional usefulness as conceptually distinct forms of usefulness in the context of sport wearable technologies. This distinction provides a clearer understanding of how different forms of usefulness are associated with user evaluations and behavioral intentions. Second, the study incorporates perceived value as a mediating construct, helping explain how different dimensions of usefulness are associated with intention to use through value-based evaluations. Third, the study adopts a hybrid analytical approach that combines PLS-SEM and fsQCA. This approach enables the simultaneous examination of linear relationships and configurational patterns, providing a more comprehensive understanding of wearable device adoption.

## 2 Literature review and hypotheses

### 2.1 Technology acceptance model and the conceptualization of exercise and functional usefulness

The Technology Acceptance Model (TAM) (13) is one of the most widely used frameworks for explaining technology adoption and use. Within TAM, perceived usefulness refers to the extent to which individuals believe that using a system will improve their performance and has consistently been identified as a key predictor of behavioral intention (13, 29). Subsequent studies have supported the importance of perceived usefulness across a range of sport-related technologies, including health applications (30), social media platforms (31), and wearable devices (32).

Despite its broad applicability, the traditional treatment of perceived usefulness as a unidimensional construct has been questioned, particularly in contexts where technologies serve multiple functions simultaneously (33, 34). This limitation is especially relevant to sport wearable devices. Unlike conventional information systems, sport wearable devices simultaneously provide exercise-related support and technical performance information (16). Treating usefulness as a single construct may therefore obscure important differences in how various forms of usefulness are associated with adoption decisions.

To address this limitation, this study distinguishes between two forms of usefulness: exercise usefulness (EU) and functional usefulness (FU). Exercise usefulness refers to users’ perceptions that a device helps them achieve exercise goals, maintain motivation, and reinforce healthy behaviors. Functional usefulness refers to users’ perceptions of the device’s technical performance, including measurement accuracy, data reliability, interface usability, and connectivity. Accordingly, this study conceptualizes EU and FU as two conceptually distinct forms of usefulness in the context of sport wearable devices. Although both forms of usefulness may be associated with wearable device adoption, they may influence perceived value and intention to use in different ways. Examining these differences may provide a more nuanced understanding of technology adoption in the context of sport wearable devices. This perspective is consistent with previous research advocating the adaptation of TAM constructs to specific technological contexts in order to better explain technology acceptance behavior (35, 36).

### 2.2 Exercise usefulness and perceived value

Perceived value refers to consumers’ overall assessment of a product or service based on a comparison between perceived benefits and costs (28). In technology adoption research, perceived value reflects users’ evaluations of whether a product’s benefits outweigh its associated costs (37). Accordingly, perceived value has been widely recognized as an important mechanism linking usefulness perceptions to behavioral intention in wearable technology research (38, 39).

In the context of sport wearable devices, exercise usefulness is expected to be positively associated with perceived value because users are more likely to value devices that support their exercise-related goals. When users perceive that a device enhances motivation, provides real-time activity feedback, and supports consistent exercise behavior, they are more likely to evaluate the device favorably and perceive its benefits as outweighing its associated costs (28). Supporting this view, Hayat et al. (40) found that usefulness related to health and exercise outcomes was positively associated with perceived value among wearable device users. Therefore, greater exercise usefulness is expected to be associated with more favorable value evaluations.

H1. Exercise usefulness is positively associated with perceived value of sport wearable devices.

### 2.3 Functional usefulness and perceived value

Functional usefulness is also expected to be positively associated with perceived value. When users perceive a device as accurate, reliable, and easy to use, they are more likely to evaluate it favorably and perceive its benefits as outweighing its associated costs (27). This argument is consistent with the means-end chain framework, which suggests that functional product attributes play an important role in consumers’ value evaluations (28).

Empirical evidence supports this relationship. Yang et al. (39) found that technical aspects of perceived usefulness were positively associated with perceived value among wearable device users. Similarly, Mathavan et al. (41) reported that the functional attributes of fitness wearables significantly predicted overall value evaluations. Therefore, greater functional usefulness is expected to be associated with more favorable value evaluations.

H2. Functional usefulness is positively associated with perceived value of sport wearable devices.

### 2.4 Perceived value and intention to use

Intention to use refers to an individual’s willingness to adopt or continue using a technology (13, 29). In wearable technology research, intention to use is commonly examined as a key outcome because it reflects users’ readiness to adopt or continue using a device (11, 36).

TAM originally identified perceived usefulness and perceived ease of use as the primary determinants of behavioral intention. More recent studies have also emphasized the importance of perceived value, which reflects users’ evaluations of benefits relative to costs (37, 39). According to the Value-Based Adoption Model (VAM), adoption intentions are shaped by users’ overall value assessments, which reflect a comparison between perceived benefits and perceived sacrifices associated with a technology (60). When users perceive greater value from a device, they are more likely to develop favorable intentions toward its adoption and continued use (27, 28).

This relationship is particularly relevant in the context of sport wearable devices, which often require both financial investment and continued engagement. Previous studies have consistently reported a positive association between perceived value and intention to use wearable devices (38, 39, 41). For example, Yang et al. (39) found that perceived value significantly mediated the relationship between usefulness perceptions and adoption intention, highlighting its role in linking technology perceptions to behavioral intention.

H3. Perceived value is positively associated with intention to use sport wearable devices.

### 2.5 Exercise usefulness and intention to use

In addition to its indirect association through perceived value, exercise usefulness is expected to be positively associated with intention to use sport wearable devices. Within TAM, perceived usefulness has consistently been identified as one of the strongest predictors of behavioral intention (13, 29). This relationship may also be understood from a goal-oriented perspective, whereby technologies that support users’ personal objectives are more likely to be associated with behavioral intention.

Exercise usefulness is expected to be directly associated with intention to use because wearable devices are often adopted to support exercise goals. Users typically report intending to use these devices to monitor physical activity, maintain motivation, and improve exercise performance (42, 43). When users believe that a device helps them achieve these goals, they are more likely to report stronger intention to use, even in the absence of extensive cost–benefit evaluations. In such cases, intention to use may be associated more directly with exercise-related motivations than with value-based evaluations (31, 44).

Previous studies support this relationship. Steel (9) reported that wearable technology users experienced declines in physical activity and increases in controlled motivation over time. Similarly, Beckett et al. (16) reported that exercise-related usefulness was among the strongest predictors of sustained wearable device engagement.

H4. Exercise usefulness is positively associated with intention to use sport wearable devices.

### 2.6 Functional usefulness and intention to use

In parallel with exercise usefulness, functional usefulness is expected to have a direct positive effect on intention to use sport wearable devices. Whereas exercise usefulness is primarily associated with motivation and goal attainment, functional usefulness is based on users’ evaluations of the device’s technical performance (44). This distinction is consistent with the hedonic–utilitarian perspective in technology acceptance research, which suggests that utilitarian perceptions influence adoption intention through performance evaluations rather than intrinsic motivation (13, 44).

In the context of sport wearable devices, functional usefulness reflects users’ perceptions of measurement accuracy, data reliability, interface usability, and device connectivity. When users perceive a device as accurate, reliable, and easy to use, they are more likely to intend to use it regardless of broader value evaluations (19). Conversely, unreliable data or poor usability may discourage continued use even when users recognize the exercise-related benefits of the device (4, 32).

Previous studies support this relationship. Yang et al. (39) found that perceived technology accuracy significantly predicted intention to use wearable fitness devices. Similarly, El-Gayar and Elnoshokaty (4) reported that tracking functions and device integration features were among the strongest determinants of continued wearable use. More recently, Salhieh (19) showed that perceived usefulness and task–technology fit each exerted significant direct effects on continued use intention among healthcare wearable users.

H5. Functional usefulness is positively associated with intention to use sport wearable devices.

### 2.7 Perceived value, intention to use, and mediation effects

Beyond its direct association with intention to use, perceived value is also expected to mediate the relationships between exercise usefulness, functional usefulness, and intention to use. This mediation mechanism is consistent with the means-end chain framework, which suggests that perceptions of product attributes influence behavioral responses through value evaluations (28). In the context of sport wearable devices, perceptions of exercise usefulness and functional usefulness may be positively associated with perceived value, which in turn may be positively associated with intention to use.

Previous studies provide support for this mechanism. Luo et al. (45) found that exercise-related smartwatch features increased perceived value, which subsequently enhanced continuance intention. Similarly, Chang et al. (61) reported that exercise-related device features enhanced perceived value and promoted continuance intention. These findings suggest that exercise usefulness may be associated with intention to use through value-based evaluations.

A similar mechanism has been observed for functional usefulness. Wang et al. (7) found that utilitarian value significantly predicted continuance intention among smartwatch and fitness wristband users. Likewise, Luo et al. (45) reported that functional device features enhanced perceived utilitarian value, which subsequently increased continuance intention. Together, these findings suggest that functional usefulness may be associated with intention to use through perceived value.

H6. Perceived value mediates the relationship between exercise usefulness and intention to use sport wearable devices.

H7. Perceived value mediates the relationship between functional usefulness and intention to use sport wearable devices.

## 3 Materials and methods

### 3.1 Research context

South Korea represents a technologically advanced context in which wearable devices and digital health technologies have rapidly diffused, supported by a highly developed information and communication technology (ICT) infrastructure and widespread adoption of smart devices. In particular, the near-universal penetration of smartphones, coupled with a high level of societal openness to emerging technologies, has positioned wearable devices as integral tools for supporting individuals’ exercise management and health promotion. Alongside these technological developments, increasing public interest in health management and physical activity has contributed to the growing adoption of wearable devices among Korean consumers. Wearable technologies are increasingly used to monitor physical activity, track exercise performance, and provide real-time feedback that supports exercise participation and self-management. Furthermore, the expanding emphasis on preventive health behaviors and active lifestyles has increased the relevance of wearable devices as tools for supporting health and fitness-related goals. Taken together, the coexistence of a technology-friendly environment and a health-oriented lifestyle makes South Korea an appropriate and analytically meaningful context for examining the roles of exercise usefulness, functional usefulness, perceived value, and intention to use in the adoption of sport wearable devices. Accordingly, this study focuses on a Korean sample to empirically investigate the factors influencing intention to use in the context of wearable technologies.

### 3.2 Data collection

Data were collected through an online survey administered by a professional survey agency, Embrain. Participants received a monetary incentive of 1,000 KRW (approximately USD 0.70) for completing the survey. A convenience sampling approach was employed, targeting Korean adults with prior experience using sport wearable devices. To ensure the relevance of the sample, a screening question was included at the beginning of the survey, and respondents without prior experience using wearable devices were excluded from the analysis. Data collection was conducted over a three-week period, from January 5 to January 26, 2026. A total of 400 responses were initially obtained, of which 322 valid responses were retained for analysis after excluding incomplete or inattentive responses. A total of 400 responses were initially gathered. Of these, 78 were excluded based on the following criteria: responses from participants who reported no prior experience using sport wearable devices (*n* = 35), incomplete responses with missing items on key constructs (*n* = 28), and straight-line response patterns in which identical scores were selected across all items (*n* = 15). The final analytic sample comprised 322 valid responses.

The sample was relatively balanced in terms of gender, with 169 male respondents (52.5%) and 153 female respondents (47.5%). The mean age of participants was 37 years (SD = 9.8). On average, respondents reported engaging in physical exercise 3.7 times per week (SD = 2.2). Regarding wearable device usage duration, 25.2% of participants reported usage of 1–6 months, 26.4% reported 6 months to 1 year, and 48.4% indicated more than one year of usage.

This study was conducted in accordance with the ethical principles of the Declaration of Helsinki. Participation was voluntary, informed consent was obtained from all participants, and no personally identifiable information was collected or retained.

### 3.3 Data analysis

To achieve a more comprehensive understanding of the relationships among the study variables, this research adopts a mixed analytical approach that combines both symmetric and asymmetric methods.

First, partial least squares structural equation modeling (PLS-SEM) was employed as the symmetric analytical technique, using SmartPLS 4.0 (62). Prior to conducting the structural model analysis, common method bias (CMB) was assessed using the full collinearity approach proposed by Kock (46). The variance inflation factor (VIF) values for all latent constructs were below the recommended threshold of 3.3, indicating that CMB was not a serious concern in this study. The adequacy of the sample size for PLS-SEM was evaluated using the inverse square root method suggested by Kock and Hadaya (47). Assuming a minimum path coefficient in the range of 0.11–0.20, the required minimum sample size for detecting statistically significant effects at the 5% significance level was approximately 155 cases. Given that the final sample consisted of 322 observations, the sample size was deemed sufficient for conducting PLS-SEM analysis. Following the two-step approach recommended by Hair et al. (25), the measurement model was first assessed in terms of reliability and validity, including internal consistency reliability, convergent validity, and discriminant validity. Exercise usefulness (EU), functional usefulness (FU), perceived value (PV), and intention to use (IU) were each specified as independent first-order reflective constructs. Subsequently, the structural model was evaluated using a bootstrapping procedure with 5,000 resamples to test the significance of path coefficients and examine the proposed hypotheses (H1–H5). In the structural model, EU and FU were included simultaneously as independent predictors of both PV and IU, allowing for an unconfounded comparison of their unique effects while controlling for multicollinearity. Prior to path estimation, VIF values were re-examined for the predictor variables in the structural model; all values were below the recommended threshold of 3.3 (EU = 1.883, FU = 2.048, PV = 1.339), confirming that multicollinearity was not a concern. In addition, the mediating role of PV in the relationships between EU and IU (H6) and between FU and IU (H7) was examined using bootstrap-based indirect effect analysis with 5,000 resamples and 95% bias-corrected confidence intervals.

Second, fuzzy-set qualitative comparative analysis (fsQCA) was employed as the asymmetric analytical technique (48). fsQCA is suitable for examining complex relational patterns and multiple configurational pathways leading to a given outcome, and it can be applied to both small and large samples (20). Unlike traditional regression-based or SEM approaches that focus on linear net effects among variables, fsQCA is grounded in the assumption of equifinality, allowing for the identification of multiple configurations of conditions associated with the same outcome (26). Prior to the fsQCA analysis, contrarian case analysis was conducted to explore the potential presence of asymmetric relationships between the outcome variable (intention to use) and its antecedents. Following the procedures outlined by Pappas and Woodside (20), fsQCA was conducted in three stages: calibration, necessary condition analysis, and sufficiency analysis. Continuous variables were calibrated into fuzzy sets based on theoretically and empirically grounded threshold values. Through this process, multiple configurational pathways explaining high levels of intention to use were identified. By integrating PLS-SEM and fsQCA, this study enables the simultaneous examination of linear relationships and nonlinear configurational patterns, thereby providing a more nuanced understanding of the patterns underlying associations with intention to use. This mixed-method analytical strategy has been increasingly adopted in social science research to enhance the robustness and explanatory power of empirical findings (49–51). Accordingly, the present study leverages the complementary strengths of both methods to improve the validity and rigor of the results.

## 4 Results

### 4.1 Measurement model assessment

To assess the reliability and validity of the measurement model, this study followed the procedures recommended by Hair et al. (25). Exercise usefulness (EU), functional usefulness (FU), perceived value (PV), and intention to use (IU) were each specified as first-order reflective constructs. Indicator reliability was assessed by examining the outer loadings of each measurement item. All items exhibited outer loadings above the recommended threshold, and therefore no indicators were removed from the analysis.

Internal consistency reliability was assessed using Cronbach’s alpha (*α*) and composite reliability (CR). All constructs demonstrated satisfactory internal consistency, with values exceeding the recommended threshold of 0.70. Convergent validity was evaluated using the average variance extracted (AVE), and all constructs met the recommended criterion of 0.50. Detailed measurement model results are presented in Table 1. Discriminant validity was assessed using both the Fornell–Larcker criterion (52) and the heterotrait–monotrait ratio (HTMT) proposed by Henseler et al. (53). The square root of AVE for each construct exceeded its correlations with all other constructs, satisfying the Fornell–Larcker criterion. All HTMT values were below the recommended threshold of 0.85, further confirming adequate discriminant validity (see Table 2). Notably, the HTMT value between EU and FU was 0.822, confirming that despite their theoretical relatedness, the two constructs are empirically distinct. Taken together, these findings indicate that the measurement model demonstrates satisfactory levels of reliability and validity.

**Table 1:** Evaluation of the measurement model.

Construct/item	Outer loading
Exercise usefulness ( α = 0.833; CR = 0.888; AVE = 0.667)	
Wearable devices help me achieve my exercise goals	0.719
Wearable devices encourage me to maintain regular exercise habits.	0.800
Wearable devices increase my motivation to exercise more.	0.880
Wearable devices help me monitor and improve my exercise performance.	0.859
Functional usefulness ( α = 0.832; CR = 0.890; AVE = 0.670)	
Wearable devices accurately track my physical activity data	0.785
Wearable devices provide reliable and precise exercise-related data that I can trust	0.831
The interface and display of wearable devices are easy to navigate and use	0.866
The customization features of wearable devices allow me to tailor the device to my needs	0.790
Perceived value ( α = 0.896; CR = 0.923; AVE = 0.707)	
The price of wearable device is reasonable.	0.807
Wearable device has good value for price	0.878
In the present price, wearable device provide good value	0.883
The material of a wearable device provides good value.	0.855
Wearable device’s hardware has good value.	0.776
Intention to use ( α = 0.938; CR = 0.948; AVE = 0.672)	
I intend to continue using the wearable device I have used most recently.	0.823
I intend to continue using the wearable device I have used most recently in the future.	0.853
I plan to continue using the wearable device I have used most recently.	0.874

*α* = Cronbach’s alpha; CR = composite reliability; AVE = average variance extracted.

**Table 2:** Discriminant validity.

Construct	EU	FU	PV	IU
EU	**0.817**	0.822	0.488	0.597
FU	0.678	**0.818**	0.567	0.595
PV	0.416	0.49	**0.841**	0.411
IU	0.517	0.52	0.372	**0.928**

Diagonal elements (bold) = square root of AVE. Upper triangle = HTMT (Heterotrait-Monotrait ratio). Lower triangle = Pearson correlations between construct scores. PV = perceived value; IU = intention to use.

### 4.2 Structural model assessment

Following the assessment of the measurement model, the structural model was evaluated in accordance with the procedures recommended by Sarstedt, Ringle, and Hair (54). Prior to path estimation, variance inflation factors (VIFs) were examined to assess potential multicollinearity. All VIF values were below the recommended threshold of 3.3, indicating that multicollinearity was not a concern (46). The significance of the path coefficients was assessed using a bootstrapping procedure with 5,000 resamples. The structural model specified EU and FU as simultaneous predictors of both PV and IU, with PV also included as a direct predictor of IU, thereby enabling the assessment of Hypotheses 1–5.

Regarding the antecedents of perceived value, both EU and FU showed significant positive associations with PV. EU was positively associated with PV (*β* = 0.172, *p* = 0.010), supporting H1, whereas FU demonstrated a comparatively stronger association with PV (*β* = 0.378, *p* < 0.001), supporting H2. With respect to the antecedents of intention to use, EU demonstrated a significant direct positive association on IU (*p* < 0.001), supporting H4. FU also showed a significant direct positive association on IU (*p* < 0.001), supporting H5. In addition, the direct association of PV on IU was statistically significant (*p* = 0.018), supporting H3. Together, EU and FU explained 26.3% of the variance in PV (*R*^2^ = 0.263), and the full model explained 33.9% of the variance in IU (*R*^2^ = 0.339).

These findings suggest that in the context of sport wearable devices, users’ intention to use is associated with both exercise-related and functional benefit perceptions through complementary patterns. While EU and FU were each directly associated with adoption intention, FU also showed a more prominent association through a value-mediated evaluative pathway, as evidenced by its comparatively stronger association on PV (*p* < 0.001) relative to EU (*p* = 0.010). Detailed results are presented in Table 3.

**Table 3:** Structural model path coefficients.

Hypothesis	Path	*β*	SE	*t*	95% CI	*p*
Outcome: perceived value (*R*^2^ = 0.263)						
H1	EU → PV	0.172	0.067	2.587	[0.042, 0.302]	0.010*
H2	FU → PV	0.378	0.067	5.688	[0.248, 0.509]	0.001***
Outcome: intention to use (*R*^2^ = 0.339)						
H3	PV → IU	0.126	0.053	2.378	[0.022, 0.230]	0.018*
H4	EU → IU	0.299	0.064	4.692	[0.174, 0.424]	0.001***
H5	FU → IU	0.253	0.066	3.830	[0.124, 0.383]	0.001***

*β* = standardized path coefficient; SE = standard error; 95% CI based on percentile bootstrap. *p < 0.05, ***p < 0.001.

### 4.3 Indirect effects and mediation analysis

To examine the mediating role of perceived value (PV), bootstrap-based indirect effect analyses were conducted using 5,000 resamples and 95% bias-corrected confidence intervals. The indirect effect of EU on IU via PV was not statistically significant (*β* = 0.019, 95% CI [−0.000, 0.047], *p* = 0.053). Accordingly, H6 was not supported. In contrast, the indirect effect of FU on IU via PV was statistically significant (*β* = 0.047, 95% CI [0.004, 0.098], *p* = 0.032), supporting H7 and indicating partial mediation. A bootstrap difference test was conducted to compare the two indirect effects. The difference was not statistically significant (Δ*β* = 0.028, 95% CI [−0.004, 0.078], *p* = 0.100), suggesting that the indirect effects of EU and FU did not differ significantly in magnitude. Detailed results are presented in Table 4.

**Table 4:** Indirect effects and bootstrap difference test.

Hypothesis	Path	*β*	SE	95% CI	*p*
Indirect effects					
H6	EU → PV → IU	0.019	0.013	[−0.000, 0.047]	0.053
H7	FU → PV → IU	0.047	0.024	[0.004, 0.098]	0.032*
Bootstrap difference test					
FU indirect − EU indirect		0.028	0.022	[−0.004, 0.078]	0.100

*p < 0.05.

### 4.4 Contrarian case analysis

A contrarian case analysis was conducted prior to fsQCA to examine whether asymmetric relationships existed between intention to use (IU) and its antecedents. Following Pappas and Woodside (20), exercise usefulness (EU), functional usefulness (FU), perceived value (PV), and IU were transformed into quintiles and analyzed using cross-tabulations. Cross-tabulations revealed contrarian cases in both directions: some cases with low EU, FU, or PV exhibited high IU, while others with high antecedent levels showed low IU (see Appendix 1). These findings indicate that the relationships among the study variables cannot be fully explained by a single linear pattern, thereby justifying the application of fsQCA.

Prior to the fsQCA analysis, all variables were calibrated into fuzzy-set membership scores following the procedure proposed by Ragin (48). The 95th percentile was used as the threshold for full membership (1), the 5th percentile as the threshold for full non-membership (0), and the crossover point (0.5) was set at 4.0 for EU, FU, and PV. For IU, the crossover point was set at the empirical median (3.77). The calibration thresholds are reported in Table 5. Following Fiss (55), cases calibrated at the crossover point were assigned a value of 0.501 to avoid ambiguity in set membership and ensure their inclusion in the analysis.

**Table 5:** Calibration thresholds for fsQCA analysis.

Concept	Full membership (95%)	Crossover point (50%)	Full non-membership (5%)
Exercise usefulness	4.99	4.00	2.75
Functional usefulness	5.00	4.00	2.75
Perceived value	4.80	4.00	3.00
Intention to use	4.66	3.77	3.00

### 4.5 Necessary condition analysis

A necessary condition analysis was conducted to examine whether any single antecedent constituted a necessary condition for high intention to use. Exercise usefulness (EU), functional usefulness (FU), perceived value (PV), and their negations were evaluated. As shown in Table 6, none of the examined conditions reached the recommended consistency threshold of 0.90. These findings indicate that no individual condition is necessary for the outcome, suggesting that high intention to use is associated with combinations of conditions rather than any single antecedent in isolation (26, 48). Accordingly, the analysis proceeded to the examination of sufficient configurations. Detailed results are presented in Table 6.

**Table 6:** Necessary condition analysis.

Condition	Consistency	Coverage
EU	0.855	0.818
~EU	0.622	0.694
FU	0.848	0.800
~FU	0.600	0.677
PV	0.874	0.865
~PV	0.597	0.647

### 4.6 Sufficiency analysis

Given the absence of necessary conditions, a sufficiency analysis was conducted using the fsQCA truth table algorithm. Following Fiss (55), a frequency threshold of 2 and a consistency threshold of 0.85 were applied, along with a minimum PRI consistency threshold of 0.50 (56). The intermediate solution was selected as the primary basis for interpretation, while the parsimonious solution was used to identify core conditions.

The analysis yielded five configurations associated with high intention to use (Table 7). The overall solution consistency was 0.895, exceeding the recommended threshold, and individual configuration consistencies ranged from 0.921 to 0.933. The overall solution coverage was 0.789, indicating that the identified configurations collectively accounted for a substantial proportion of cases with high intention to use. Although several configurations exhibited negative unique coverage values, this is not uncommon in fsQCA and likely reflects overlap among configurations (57). The largest configurations were Solution 2 (EUFU~PV; *n* = 81) and Solution 1 (EUFUPV; *n* = 41), together accounting for 122 of the 322 cases. Across the identified solutions, functional usefulness appeared in four of the five configurations, whereas exercise usefulness appeared as a core condition in Solutions 1 and 2. Perceived value was present in some configurations and absent in others, indicating a more context-dependent role. Solution 1 combined EU, FU, and PV. Solution 2 combined EU and FU in the absence of PV. Solution 3 was defined primarily by the presence of FU. Solution 4 combined FU and PV, whereas Solution 5 was characterized by the presence of PV. Together, these configurations demonstrate that high intention to use can emerge from multiple alternative combinations of conditions.

**Table 7:** fsQCA results (configurations leading to high intention to use).

Conditions	Sol1	Sol2	Sol3	Sol4	Sol5
EU	⬤	⬤			
FU	⬤	⬤	●	●	
PV	●	⊗		●	●
*n*	41	81	53	8	3
Raw coverage	0.652	0.479	0.362	0.378	0.337
Unique coverage	0.077	−0.253	−0.416	−0.403	−0.431
Consistency	0.931	0.931	0.921	0.933	0.925

Solution coverage = 0.789, solution consistency = 0.895, frequency cutoff = 2, consistency cutoff = 0.85. Black circles (●) represent the presence of causal conditions. Circles with “x” (⊗) represent the absence or negation of causal conditions. Larger circles represent core conditions, while smaller circles mean peripheral conditions. Blank spaces indicate the condition does not matter for the outcome.

Overall, the findings support the principle of equifinality by identifying multiple pathways associated with high intention to use. The results also illustrate causal asymmetry, as the same conditions assume different roles across configurations. Robustness analyses using alternative calibration thresholds and consistency cutoffs produced substantively similar results (Table 8), providing evidence for the robustness of the configurational solutions.

**Table 8:** fsQCA robustness check results.

Specification	Freq. Cutoff	Cons. Cutoff	Matching configurations	Solution coverage	Solution consistency
Baseline calibration (EU/FU/PV = 4.00; IU = 3.77)	2	0.85	7/8	0.721	0.871
Crossover −5% (EU/FU/PV = 3.80; IU = 3.58)	2	0.85	7/8	0.759	0.897
Crossover +5% (EU/FU/PV = 4.20; IU = 3.96)	2	0.85	6/8	0.645	0.861
Consistency threshold = 0.80	2	0.80	7/8	0.721	0.871
Consistency threshold = 0.90	2	0.90	4/8	0.565	0.914
Frequency threshold = 3	3	0.85	7/8	0.721	0.871

### 4.7 Robustness check

As presented in Table 8, four of the five alternative specifications produced configurational solutions that were largely consistent with the baseline model, retaining seven of the eight original configurations associated with the outcome. Across these specifications, solution coverage ranged from 0.645 to 0.759, while solution consistency ranged from 0.861 to 0.914. Minor deviations were observed when the crossover point was increased by 5% (6/8 configurations retained) and when the consistency threshold was raised to 0.90 (4/8 configurations retained). These changes are consistent with the expected effects of applying more conservative calibration and sufficiency criteria and do not indicate substantive instability in the overall solution structure. Collectively, the findings provide evidence for the robustness of the configurational solutions reported in Table 8.

## 5 Discussion

This study investigated the mechanisms underlying intention to use toward sport wearable devices by extending the Technology Acceptance Model (TAM). Specifically, perceived usefulness was reconceptualized as a multidimensional construct comprising exercise usefulness and functional usefulness, and perceived value was incorporated as a mediating variable within the research model. A mixed-method approach combining symmetric analysis (PLS-SEM) and asymmetric analysis (fsQCA) was employed to examine both the linear net effects of each construct and the complex configurational pathways through which they combine to influence intention to use. The findings provide a comprehensive understanding of how different dimensions of usefulness operate through distinct pathways to influence intention to use. In the following sections, the theoretical and practical implications are discussed based on the key results derived from both symmetric and asymmetric analyses.

### 5.1 Discussion of symmetric analysis findings

The PLS-SEM results provide support for all five direct path hypotheses (H1–H5) and one of the two mediation hypotheses (H7), offering a nuanced picture of how exercise usefulness (EU) and functional usefulness (FU) independently shape perceived value (PV) and intention to use (IU) among sport wearable device users.

Regarding the antecedents of perceived value, both EU and FU were positively associated with PV, supporting H1 (*p* < 0.05) and H2 (*p* < 0.001) respectively. However, FU exerted a substantially stronger effect on PV (*β* = 0.378) than EU (*β* = 0.172). This pattern is consistent with the means-end chain framework (28), which posits that functional product attributes serve as primary reference points in consumers’ cost–benefit evaluations. Users place greater emphasis on the device’s technical performance, including measurement accuracy, interface usability, and data precision, than on exercise-related benefits when evaluating its value. This is consistent with prior research demonstrating that functional attributes of wearable devices are stronger predictors of perceived value than experiential or hedonic perceptions (39, 41). With respect to the direct antecedents of intention to use, EU demonstrated a stronger direct association with IU (H4; *β* = 0.299, *p* < 0.001) than FU (H5; *β* = 0.253, *p* < 0.001). This pattern is broadly consistent with prior research suggesting that experiential and goal-related benefits tend to be more directly associated with behavioral intention than functional evaluations (44). When users perceive that a device directly supports their exercise goals, higher intention to use appears to be associated with this perception without requiring an intervening evaluative process. Patel et al. (43) similarly argued that perceived alignment between device features and personal health objectives is a critical determinant of continued engagement motivation. PV was also positively associated with IU (H3; *β* = 0.126, *p* < 0.05), suggesting that favorable cost–benefit evaluations show an independent association with adoption intention, albeit with a modest effect.

The mediation analysis revealed an asymmetric pattern that reflects the distinct roles of exercise usefulness (EU) and functional usefulness (FU). The indirect effect of FU on intention to use (IU) through perceived value (PV) was significant (H7; *p* < 0.05), suggesting the presence of a value-mediated pathway. In contrast, the indirect effect of EU on IU through PV did not reach statistical significance (H6; *p* = 0.053). The non-significant mediation of PV in the EU–IU relationship (H6) warrants theoretical attention. This finding may be explained by the usage stage effect: the present sample comprises current wearable device users who have already passed the initial adoption threshold. Prior research on technology continuance suggests that perceived value plays a more prominent role during initial adoption decisions than during continuance stages, with direct utility perceptions becoming more decisive as users accumulate device experience (58, 59). Rather than indicating a theoretical weakness, the non-significant mediation effect of EU may provide additional insight into how exercise-related perceptions influence intention to use. Specifically, EU appears to be more directly associated with intention to use, with a comparatively limited role for value-based evaluations. This interpretation is consistent with research suggesting that intrinsically motivated technology use is often linked to behavioral intention through goal attainment and motivational processes rather than through explicit cost–benefit assessments (27, 44).

The bootstrap difference test indicated that the indirect effects of EU and FU did not significantly differ in magnitude (*p* = 0.100). This finding suggests that the value-mediated contributions of the two usefulness dimensions were broadly comparable, despite differences in statistical significance One possible explanation is that the sample consisted exclusively of current wearable users, who may already hold relatively favorable perceptions of exercise-related benefits and therefore rely less on value evaluations when forming intention to use. Furthermore, all findings in the present study pertain to intention to use rather than observed behavioral engagement, and the public health implications of adoption intention should therefore be interpreted as preliminary pending longitudinal behavioral evidence.

### 5.2 Discussion of asymmetric analysis findings

The fsQCA analysis complements and extends the PLS-SEM findings by identifying five sufficient configurational pathways to high intention to use, collectively demonstrating equifinality that is, multiple distinct combinations of conditions can produce the same outcome. This perspective reveals aspects of the data that symmetric analysis alone cannot capture, particularly the contingent and combinatorial nature of adoption motivation.

Functional usefulness emerged as the most central condition across configurations, appearing as a core or peripheral condition in four of the five solutions (Sol1–Sol4). This prominence of FU across multiple pathways reinforces and extends the PLS-SEM finding of a significant FU–IU direct path, and suggests that functional performance perceptions constitute a nearly indispensable correlate of adoption intention regardless of the specific configuration in which they appear. Exercise usefulness, in contrast, functioned as a core condition in Sol1 and Sol2, indicating that its decisive role is most pronounced when combined with functional usefulness. This configurational pattern aligns with the direct pathway dominance of EU identified in the symmetric analysis, while also revealing that EU’s contribution is most impactful in the context of broader multi-condition constellations.

The five solutions can be broadly interpreted as reflecting distinct adoption profiles Solution 1 (Sol1) represented the most comprehensive pathway, characterized by the simultaneous presence of all three conditions. This configuration suggests that users who perceived exercise benefits, functional performance, and positive value evaluations concurrently exhibited the highest likelihood of adoption intention. Sol2, the largest configuration, suggests that strong exercise-related and functional utility perceptions were associated with high intention to use even in the absence of favorable value evaluations. This finding indicates that positive perceptions of both exercise-related and functional benefits may be associated with intention to use regardless of perceived value in certain configurational contexts. Sol3 represents a more streamlined configuration in which functional usefulness was present in a pathway associated with high intention to use. This finding suggests that favorable perceptions of device performance may be associated with intention to use in some configurational contexts, even when exercise usefulness and perceived value are not consistently represented. Sol4 shows that configurations characterized by functional usefulness and perceived value were associated with high intention to use, even when exercise usefulness was not prominently represented. Sol5 represents a configuration in which perceived value was present in a pathway associated with high intention to use, suggesting that favorable value evaluations may play an important role in certain configurational contexts.

Importantly, the fsQCA results also illuminate the role of PV in ways that the symmetric analysis could not fully capture. While PV’s direct effect on IU in the PLS-SEM model was statistically significant but comparatively modest, the configurational analysis suggests that PV functions as a complementary condition within specific pathways rather than as a dominant independent driver. PV was present in three sufficient configurations associated with high IU (Sol1, Sol4, and Sol5), whereas it was not required in the highest-coverage configuration (Sol2). This distinction between PV as a significant but comparatively weaker predictor in the symmetric model and PV as a contingent configurational enabler illustrates the value of causal asymmetry as an analytical lens. The conditions associated with high IU are not simply the inverse of those associated with low IU, and net-effect approaches may underrepresent the contribution of variables whose influence depends on their combination with other conditions rather than their independent additive effects.

The integrated findings from both analytical approaches confirm that sport wearable adoption intention is shaped by a complex, multidimensional, and asymmetric causal structure PLS-SEM identifies the average linear associations of EU, FU, and PV with IU and reveals a dual-pathway pattern in which EU is associated primarily with a direct relationship to IU, whereas FU is associated with both direct and value-mediated relationships. However, these findings relate to intention to use rather than observed behavioral outcomes. fsQCA reveals that these mechanisms are embedded within distinct configurational profiles that reflect the heterogeneity of users’ adoption motivations. Together, the two methods provide a more complete understanding of sport wearable adoption than either approach could offer alone, consistent with the methodological complementarity advocated by Rasoolimanesh et al. (51) and Hasan and Bao (50).

### 5.3 Practical implications

The findings of this study offer preliminary practical implications for healthcare practitioners and digital health program designers working with current wearable device users. Given that the sample consists of Korean adults with prior wearable experience, the following implications are most directly applicable to programs targeting existing users and should be interpreted cautiously when extended to non-user populations, older adults, or non-Korean cultural contexts.

First, practitioners should consider both exercise-related goal alignment and technical performance when recommending wearable devices. The PLS-SEM results indicate that exercise usefulness and functional usefulness were both positively associated with intention to use, suggesting that users value not only the technical capabilities of a device but also its perceived relevance to their personal exercise goals. Accordingly, practitioners such as exercise specialists, community health coordinators, and primary care providers may benefit from helping individuals understand how specific device functions relate to their health and exercise objectives. For example, onboarding sessions could include collaborative goal-setting activities and demonstrations of how device features support those goals. Such approaches may be associated with stronger perceptions of device relevance among current users, though their effectiveness for non-users or those with limited device familiarity remains to be examined.

Second, public health programs may benefit from complementing device provision with strategies that enhance users’ perceptions of functional value. The findings suggest that functional usefulness was associated with intention to use both directly and indirectly through perceived value. This pattern indicates that favorable perceptions of technical performance may be more strongly associated with intention to use when accompanied by positive value evaluations. Consequently, wearable-based health promotion programs should consider incorporating post-distribution support activities, such as user education, guided interpretation of device-generated data, and ongoing feedback mechanisms. These efforts may help current users recognize the practical benefits of wearable devices and strengthen their overall value perceptions, which may in turn support sustained intention to use. However, longitudinal research is needed to determine whether stronger intentions ultimately translate into actual behavioral engagement. In addition, the extent to which such strategies apply to individuals without prior wearable device experience remains an important question for future research.

Third, the fsQCA findings highlight that high intention to use may be associated with multiple configurational pathways rather than a single dominant pattern. This finding is consistent with the precision public health perspective, which recognizes that different groups may respond to health interventions in different ways. Notably, functional usefulness appeared across most identified configurations, suggesting that perceptions of technical performance are frequently associated with intention to use. However, the configurations also demonstrate that exercise usefulness and perceived value may assume different roles depending on the broader combination of conditions. Rather than supporting a single uniform intervention approach, these findings suggest that programs targeting current wearable users in similar technological contexts may benefit from considering the diversity of factors associated with intention to use. Whether these configurational profiles generalize to non-users or populations with lower digital literacy remains an open empirical question. Future intervention programs may therefore consider assessing users’ exercise goals, value perceptions, and technology-related experiences when designing support strategies.

Fourth, digital health equity should be considered an important component of wearable-based public health initiatives. The present study focused on Korean adults with prior wearable device experience, representing a population with relatively high technological access and digital familiarity. The identified configurations therefore reflect adoption intention patterns among relatively technology-engaged users and may not capture the barriers or motivational profiles of non-users, older adults, or individuals with limited digital literacy. These populations may nonetheless stand to benefit substantially from wearable-supported physical activity programs, and future research should explicitly examine their adoption pathways. Program designers considering broader deployment should treat the present findings as a starting point rather than a generalizable blueprint, and complementary studies targeting underrepresented groups would be needed to inform equitable implementation strategies.

### 5.4 Limitations and future directions

The boundary conditions of this study should be clearly delineated. The findings apply most directly to Korean adults with prior sport wearable device experience and should not be generalized to non-users, individuals in the initial stages of adoption, older adults with lower levels of digital literacy, or populations in different cultural or institutional contexts. Despite its contributions, this study has several limitations that point to directions for future research. First, the sample was drawn from Korean adults with prior wearable experience through an online survey panel, which may limit generalizability. Cross-cultural replication studies, including Western and developing-country samples, as well as studies targeting non-users and underrepresented demographic groups, would help evaluate the robustness of the multidimensional usefulness model across contexts. Second, the cross-sectional research design constrains causal inference and prevents the study from capturing how the relative salience of EU, FU, and PV changes across stages of device use. Moreover, the study measures intention to use rather than actual device usage or physical activity behavior. Although intention is widely regarded as an important proximal predictor of behavior in technology acceptance research, the intention–behavior gap is well documented in the health behavior literature. Future research should therefore incorporate behavioral outcome measures, such as objective device usage logs or accelerometer-based physical activity data, to examine whether the intention patterns identified in this study are associated with sustained device use and subsequent behavioral outcomes. Prior research indicates that technology acceptance determinants shift over time, with utilitarian factors more prominent in early stages and experiential factors becoming more important during continuance (59). Longitudinal designs would allow future research to trace these temporal dynamics across the user lifecycle. Third, the fsQCA analysis included only three antecedent conditions EU, FU, and PV, to maintain analytical parsimony and interpretability. Incorporating additional conditions such as social influence, health consciousness, perceived privacy risk, and technology readiness may reveal more complex and differentiated causal pathways. Future configurational analyses should expand the condition set to enhance the explanatory scope of the model and further illuminate the heterogeneity of wearable adoption profiles.

## Data Availability

The raw data supporting the conclusions of this article will be made available by the authors, without undue reservation.
